# Heterozygosity in NPC may be associated with neurologic and systemic phenotypes

**DOI:** 10.3389/fneur.2025.1618380

**Published:** 2025-08-12

**Authors:** Tatiana Brémovà-Ertl, Sabina Tahirovic, Silva Katušić Hećimović, Kyriakos Martakis, Marianne Rohrbach, Matthias Gautschi, Radhika Dhamija, Jaya Ganesh, Melinda Peters, Mark Walterfang, Susanne A. Schneider

**Affiliations:** ^1^Department of Neurology, Department of Neuropediatrics and Center for Rare Diseases, University Hospital Inselspital, Bern, Switzerland; ^2^German Center for Neurodegenerative Diseases (DZNE) e.V., Munich, Munich, Germany; ^3^Division of Molecular Medicine, Ruđer Bošković Institute, Zagreb, Croatia; ^4^Klinik für Neuropädiatrie, Epileptologie und Sozialpädiatrie der Justus-Liebig Universität Giessen und des Universitätsklinikums Gießen-Marburg, Giessen, Germany; ^5^Department of Pediatrics, Medical Faculty and University Hospital, University of Cologne, Cologne, Germany; ^6^Referenzzentrum hereditärer Stoffwechselstörungen, Universitäts-Kinderspital Zürich, Zürich, Switzerland; ^7^Swiss Reference Centre for Inborn Errors of Metabolism, Site Bern, INSELSPITAL, Department of Paediatrics, Division of Paediatric Endocrinology, Diabetology and Metabolism, University Hospital Bern, Julie-von-Jenner Haus, Bern, Switzerland; ^8^Departments of Clinical Genomics and Neurology, Mayo Clinic Rochester, Rochester, MN, United States; ^9^Department of Genetics and Genomic Sciences, Icahn School of Medicine at Mount Sinai, New York, NY, United States; ^10^Division of Genetics and Genomics, Boston Children’s Hospital, Boston, MA, United States; ^11^Department of Psychiatry, Florey Institute of Neuroscience and Mental Health, University of Melbourne, Parkville, VIC, Australia; ^12^INSPIRE-PNRM+, Neuroimaging Center (NIC), University Medical Center of the Johannes Gutenberg University, Mainz, Germany

**Keywords:** Niemann-Pick disease type C, carrier, heterozygosity, heterozygote, rare disease

## Abstract

**Background:**

Niemann-Pick disease type C (NPC) is a pan-ethnic, progressive, recessively inherited lysosomal disorder that affects 1:100,000 live births. Emerging biochemical, genetic, and clinical evidence challenges the traditional view that disease-associated variants in the genes associated with the typical phenotype NPC manifest as an exclusively autosomal recessive disorder. While biallelic pathogenic variants cause the NPC disease phenotype, heterozygous carriers may exhibit phenotypic traits attributable to a partial loss of *NPC1* or *NPC2* function.

**Methods:**

We conducted a literature search of articles relevant to heterozygosity in NPC genes and genes associated with other lysosomal diseases. A narrative mini-review format was employed with the intention of providing a brief overview of the frequency of NPC carriers, as well as the biochemical, genetic, non-clinical, and clinical evidence available for readers seeking to understand the scientific basis for why NPC heterozygosity should be discussed and considered as a potential risk factor for the development of neurological phenotype or neurodegenerative diseases.

**Conclusion:**

Heterozygosity for many genes, including *NPC1* variants, (“carriers” of a single variant in an NPC gene) can be clinically consequential. Recognizing the effects of *NPC1* heterozygosity has profound implications for diagnosis, clinical monitoring, and potential early intervention. By broadening our understanding of the genetic and phenotypic spectrum of NPC, we can improve detection (which is straightforward in obligate heterozygotes, i.e., parents of NPC patients), reduce long-term health risks, and utilize targeted treatments that address the needs of carriers as well as affected individuals.

## Introduction

Niemann-Pick disease type C (NPC) is a pan-ethnic, progressive, recessively inherited lysosomal disorder that affects 1:100,000 live births. Emerging biochemical, genetic, and clinical evidence challenges the traditional view that disease-associated variants in the genes associated with the typical phenotype NPC manifest as an exclusively autosomal recessive disorder. While biallelic pathogenic variants cause the classical NPC disease phenotype, heterozygous carriers may exhibit phenotypic traits attributable to a partial loss of *NPC1* or *NPC2* function (1, 2).

In September 2024, the US Food and Drug Administration approved two novel treatments for NPC, each which target different pathologies of the disease [levacetylleucine (AQNEURSA™)2024 (3), and arimoclomol (MIPLYFFA™)2024—authorized in combination with miglustat (4)]. In Europe (and other countries worldwide) miglustat (ZAVESCA™) is authorized as a treatment for NPC. The availability of approved treatments for patients with NPC has renewed interest in an ongoing question of whether heterozygous carriers of a single NPC disease-associated variant (e.g., parents, grandparents and siblings) are at risk of developing neurological diseases in older age, potentially converging with the manifestations of the biallelic early onset disease.

A single (heterozygous) pathogenic variant in a gene associated with a recessive disorder (including NPC) has been associated with subclinical abnormalities and an increased risk of developing the disease or posing a significantly increased risk for late-onset neurodegeneration (5). There are already several well-established associations between neurodegenerative disorders and heterozygosity for several genes of varying function, including *APOE4* (6), *TREM2*, *ABCA7* and *SORL1* (7) and Alzheimer’s disease (AD), amyotrophic lateral sclerosis (ALS)/frontotemporal dementia (8), and *LRRK2* and Parkinson’s disease (PD) (9). In other lysosomal disease, heterozygosity for specific variants in glucocerebrosidase (GBA) predisposes to PD (10).

Heterozygotes for NPC disease-associated variants (traditionally referred to as “carriers”) have been reported with significant neurological signs and symptoms (late-onset neurological diseases and neuropsychiatric symptoms) (1, 11). Though the clinical presentation may be subtler than in homozygotes or compound heterozygotes, the high prevalence [carrier frequency calculated as 1 in 159 (12)] of NPC heterozygotes implies a high, underreported, and unmet medical need, including potential treatment implications, i.e., allowing for early treatment to reduce or even prevent long-term health complications as these heterozygous individuals age. Schneider and colleagues previously summarized clinical observations in human and animal NPC carriers. However, given that NPC heterozygotes may benefit from recently approved NPC-targeted therapies, particularly those related to lysosomal or mitochondrial dysfunction, an updated review and summary is warranted to reflect the latest research into the neurological and systemic phenotypes of NPC carriers.

## Methods

We conducted a literature search in PubMed of articles relevant to heterozygosity in NPC genes and genes associated with other lysosomal diseases. The search terms are available in the (5) A. Bibliographies of the main review papers were also used to detect other relevant articles. A narrative mini-review format was employed with the intention of providing a brief overview of the frequency of NPC carriers, as well as the biochemical, genetic, non-clinical, and clinical evidence available for readers seeking to understand the scientific basis for why NPC heterozygosity should be discussed and considered as a potential risk factor for the development of neurological phenotype or neurodegenerative diseases, and the rationale behind therapeutic intervention for carriers.

### Carrier frequency

The incidence of heterozygous carriers can be determined from the Hardy–Weinberg law. If NPC is a recessive trait expressed at a frequency of 1:100,000, then carrier frequency is 1 is 159 (calculated from an online calculator: https://www.perinatology.com/calculators/Hardy-Weinberg.htm), with this carrier frequency, even a small percentage of carriers showing mild symptoms could represent a significant number of individuals affected at a population level (12).

### Biochemical basis

Niemann-Pick disease type C is characterized by endosomal-lysosomal accumulation of multiple lipid cargoes, including unesterified cholesterol and glycosphingolipids (13–15). Pathologically, there is evidence of storage of these lipids in neurons, lymphoid tissue, bone marrow and the liver and spleen, leading to profound neurological and systemic dysfunction (16–18). The *NPC1* and *NPC2* genes encode interacting proteins essential for intracellular trafficking of cholesterol, glycosphingolipids and other lipids from the late endosomes/lysosome to the endoplasmic reticulum (ER). NPC exhibits marked phenotypic variability, even in some multiplex kindreds, invoking possible effects of modifier genes, sex and environmental factors, in addition to the functional consequences of *NPC1* or *NPC2* pathogenic variants *per se* (19).

Even partial loss of function due to heterozygosity is shown to impair lipid export, leading to intracellular lipid accumulation (20, 21). While not as pronounced as in homozygotes or compound heterozygotes, this dysregulation can contribute to subclinical or overt abnormalities in lipid metabolism that could progress with ageing (11). Elevated oxysterol levels, reflecting oxidation of sequestered unesterified cholesterol, have been observed in some carriers (1), suggesting that even one defective *NPC1* allele can have physiological consequences.

### Genetic basis

It is increasingly evident that one functional copy of the *NPC1* or *NPC2* gene is not sufficient to prevent age-related cellular dysfunction. Certain *NPC1* variants may exert dominant-negative effects, where the mutant allele interferes with the function of the normal allele (11). This could explain why some heterozygotes exhibit measurable clinical or biochemical phenotypes (22). In some individuals, heterozygosity for *NPC1* variants combined with other genetic or environmental factors may serve as a second “hit” and exacerbate symptoms, leading to clinical manifestations like those seen in other recessive disorders (19). More complex, non-Mendelian mechanisms, such as synergistic heterozygosity [reviewed by (23)], other forms of digenic inheritance (24) and mosaicism, may contribute to the potential pathogenicity of *NPC1* variants.

### Animal studies

NPC can be modelled effectively in a spontaneous mutant mouse model (*Npc1−/*−) (25) which features a range of the biochemical (26), pathological, neurological, and behavioral characteristics of NPC disease in humans, including the storage of unesterified cholesterol, bis(monoacylglycero)phosphates (BMPs) and sphingolipids, neuroinflammation (27), Purkinje cell loss, axonal swellings, neuronal vacuoles, impaired glucose metabolism, and progressive motor impairments such as tremor, ataxia, and progressive body weight loss (18, 28). Notably, studies in heterozygous *NPC1* knockout mice (*Npc1+/−*) have revealed that these mice also feature biochemical histopathological, and neurological features as the null mouse, including cholesterol accumulation, lysosomal and mitochondrial dysfunction, Purkinje cell loss and increased tau phosphorylation.

The cerebral cortex in aged *Npc1+/−* brains shows decreased cholesterol levels in lipid rafts, and reductions in adenosine triphosphate (ATP) levels as well as decreased mitochondrial ATP synthase activity, as compared with those in the *Npc1+/+* wild type controls. It has previously been described that the accumulation of cholesterol negatively impacts mitochondrial function and may contribute to neurodegeneration in NPC. In particular, impaired cholesterol trafficking and the resultant intracellular cholesterol accumulation result in an increased cholesterol level in mitochondria, which induces mitochondrial dysfunction, thereby affecting ATP synthase activity and reducing cellular ATP levels (29). Mitochondrial dysfunction with energy depletion causes cell death (30), and there is substantial evidence that mitochondrial dysfunction triggers neurodegenerative diseases, including AD, PD, and ALS (31).

That heterozygous *NPC1* mouse variants are associated with impaired neuronal functions, including mitochondrial dysfunction and tau abnormalities, which synergistically cause neurodegeneration in the *Npc1+/−* mouse brains reinforces the findings that human heterozygous *NPC1* variants may be a risk factor for neurodegenerative disorders, such as synucleinopathy or tauopathy, in the aged population (20, 29).

### Clinical observations in heterozygous carriers

Individual case reports, and small case series document associations between NPC heterozygosity and neurological symptoms. These include heterozygosity for *NPC1* and tremor (32), progressive supranuclear palsy (PSP—associated with an *NPC2* splicing variant), (33), parkinsonism (34, 35) and Alzheimer’s disease (36). Lopergolo et al. (36) described a kindred of five living siblings, who all expressed the *c.3034G>T (p. Gly1012Cys)* variant in *NPC1*, and who showed elevated oxysterols, in addition to markers of Alzheimer’s disease, including increased amyloid positron emission tomography (PET) burden in cerebral cortex and cerebral hypoperfusion on PET. Heterozygous carriers may also display lipid profile irregularities, such as elevated low-density lipoprotein (LDL) derived cholesterol or altered plasma lipids, pointing to a systemic impact of reduced NPC1 function (2, 37, 38).

Cupidi et al. (11) conducted an observational study on 50 patients with neurodegenerative dementia; most patients had a ‘dementia plus’ presentation. Four patients were found to be heterozygous for *NPC1* variants, including c*.852delT, c.665A > G, and c.88G > A and c.441 + 1G > A,* in *NPC2*. Of note, three of four patients showed vertical supranuclear gaze palsy (VSGP), two had a history of delayed milestones, two had psychiatric manifestations, and one had gelastic cataplexy (this was the only patient lacking VSGP).

Kågedal et al. (39) found that the expression of *NPC1* at the messenger RNA (mRNA) and protein levels was elevated in the hippocampus and frontal cortex in AD patients compared to controls. In the cerebellum, which is relatively spared in AD, no difference in NPC1 expression was detected. However, *in vitro* studies showed that *NPC1* levels were not changed upon amyloid precursor protein (APP) overexpression of Aβ oligomer treatment, suggesting that altered expression of *NPC1* in AD is likely not due to key AD pathological features, but may be linked to altered homeostasis of cholesterol or other lipids, a feature that has been demonstrated in AD as well as in other neurodegenerative disorders (40). Although *NPC1* heterozygosity was not analyzed in this cohort, it demonstrates the link between altered lipid metabolism in AD and altered NPC1 function.

Benussi et al. (41) studied the manifestations associated with *NPC1* variants in two families comprising monozygotic twins homozygous for *NPC1 p. Pro888Ser*, four compound heterozygous patients *[p. Glu451Lys and p. Gly992Trp], 10 p. Pro888Ser heterozygotes, one p. Glu451Lys* heterozygote and 11 family members lacking either of these variants. The investigators assessed executive function, plasma oxysterols, and the function of cholinergic circuits by transcranial magnetic stimulation. The findings included nonsignificant elevation of oxysterols in carriers compared to noncarriers, impaired executive function in patients and carriers compared to noncarriers and increased short latency afferent inhibition in patients and carriers compared to noncarriers. There was also a correlation between oxysterol levels and certain executive and neurophysiological measures.

Bremova-Ertl et al. (1) researched the long-term health outcomes of individuals with heterozygous pathogenic variants in *NPC1* and revealed manifestations of NPC, such as metabolic, neurodegenerative and psychiatric conditions, highlighting a spectrum of phenotypic effects. Motor function, cognition, mood, sleep, and smell function were assessed in 20 first-degree heterozygous relatives of NPC. These NPC heterozygotes recapitulated characteristic features of symptomatic NPC disease and demonstrated the ocular motor abnormalities typical of NPC. Hepatosplenomegaly (71%) and increased levels of cholestane triol (33%) and plasma chitotriosidase (17%) were present. They also showed signs seen in other neurodegenerative diseases, including hyposmia (20%) and REM sleep behavior disorder (RBD) using validated questionnaires (24%) and sleep studies [Personal unpublished data (SAS)]. Cognitive function was frequently impaired, especially affecting visuo-constructive function, verbal fluency, and executive function. In terms of imaging biomarkers, FDG-PET imaging revealed significantly decreased glucose metabolic rates in 50% of participants, affecting cerebellar, anterior cingulate, parieto-occipital, and temporal regions, including one with bilateral abnormalities.

### Risk for developing neurodegenerative disorders

Erickson et al. (42) studied the association of sporadic late-onset Alzheimer’s disease (SLAD) and *NPC1* variants in a Polish population, comprising 96 subjects with AD, 152 centenarians and 120 control subjects. Three single-nucleotide polymorphisms (SNPs) in *NPC1* were evaluated; two were non-synonymous and one was intronic. These investigators found that the two non-synonymous SNPs showed allele frequencies with gradients that varied from centenarians through normal controls to SLAD. In addition, the intronic SNP was not in Hardy–Weinberg equilibrium and differed between centenarians and controls or SLAD subjects. The findings were interpreted as supporting a role for *NPC1* in aging.

Kresojević et al. (43) hypothesized that heterozygous mutations in *NPC1* may act as an independent risk factor for AD, given the biochemical (44, 45) and neuropathological overlap between the two disorders, demonstrated in both human (46) and animal studies (29, 47, 48). The authors cited an observational study performed in families recruited through the National Niemann-Pick Disease Foundation (43). Of the 57 families surveyed, 29 reported a family member with a neurodegenerative disease, including 17 cases of AD in 14 families, 8 cases of ALS in 6 different families, 8 cases of PD, 4 cases of multiple sclerosis in 3 different families, 2 cases of Huntington disease in one family and one case of multiple system atrophy (MSA). Several families reported more than one neurodegenerative disease. No diagnostic or molecular sequencing data were reported for these kindreds (43).

Three studies have studied reported the frequency of *NPC1* variants in cohorts of neurodegenerative disease patients and controls. In the first of these, Zech et al. (49), investigated patients with PD, frontotemporal lobar degeneration, and PSP. They screened the coding regions of *NPC1* and *NPC2* for genetic variants in a homogeneous German sample of patients clinically diagnosed with PD (563 subjects), frontotemporal lobar degeneration (133 subjects) and PSP (94 subjects), and compared them to 846 population-based controls. Disease-associated variants in *NPC1* or *NPC2* were found in six PD patients (1.1% of the cohort) and seven control subjects (0.8%), but not in frontotemporal lobar degeneration or progressive supranuclear palsy. All rare variants were detected in the heterozygous state, and no compound heterozygotes were reported. Although no association was found between the three studied disorders and variants in NPC genes, the authors noted that a role for heterozygous pathogenic variants of the *NPC1* and *NPC2* genes could not be ruled out as contributing to risk for age-related neurodegenerative disorders, particularly given the very homogeneous population that was studied, and the relatively small size of the population, given the rarity of different variants of the NPC genes.

Ouled Amar Bencheikh et al. (50), reported full sequencing of the coding regions of the *NPC1* gene in 2657 PD patients and 3,647 controls in three distinct cohorts. They identified 9 common variants and 126 rare variants across the three cohorts. They did not find an association between either common or rare variants of *NPC1* with PD. The authors noted several limitations of the study, including imbalances between Parkinson’s disease patients and controls in sex and age, as well as homogeneous populations that were studied, including French Canadians and Ashkenazi Jews. Like the earlier study, it cannot be ruled out that very rare disease-associated variations in *NPC1* may be associated with Parkinson’s disease. In addition, variants involving introns could not be ruled out in this study.

In 2023, Somerville et al. (51) reported on controlled studies of the frequency of *NPC1* variants in three synucleinopathies, specifically PD, RBD, and Dementia with Lewy bodies (DLB) in European cohorts. The study populations included 1,084 cases of RBD with 2,945 controls, 2,852 cases of PD with 1,686 controls, and 2,610 cases of DLB with 1920 controls. The authors identified 17 rare *NPC1* variants across all three cohorts; none showed associations individually or when grouped together.

The authors recognized several limitations of the study, including differences in age and sex between patients and controls across all three cohorts in this study, and the exclusive European ancestry of the participants. As noted in earlier studies, this investigation cannot rule out the role of very rare *NPC1* variants in alpha-synucleinopathies or the role of variants which were not detected in these cohorts.

### Risk for developing systemic disorders

Considering its centrality to endo-lysosomal trafficking and function and downstream effects on mitochondria, it is not surprising that dysfunction of the *NPC1* gene might also have systemic consequences. In homozygotes or compound heterozygotes, these effects are likely masked by severe neurologic manifestations. *NPC1* heterozygosity is associated with weight gain, insulin resistance, and obesity in experimental murine studies and several human genome-wide association studies (52). Single nucleotide polymorphisms in the *NPC1* gene in *NPC1+/+* and *NPC1+/−* subjects have been associated with obesity, body fat mass variations, dyslipidemia, insulin resistance, and type 2 diabetes in humans and mice (53). These authors have suggested that such effects could be mediated by altered blood lipid levels or abnormal steroid hormone synthesis.

## Discussion

Heterozygosity for many genes, including *NPC1* variants, (“carriers” of a single variant in an NPC gene) can be clinically consequential. Though the clinical presentations are subtler than in homozygotes or compound heterozygotes, heterozygotes for these variants may be at risk of neurologic and metabolic complications, manifesting as neurodegenerative and systemic disorders. These findings challenge the traditional view of NPC and other diseases as strictly autosomal recessive disorders that have no impact on carriers; the contrasting findings between individual case reports and series, and larger scale genetic screening studies, imply that the expression of NPC heterozygosity is not consistent with a simple Mendelian mechanism, but is likely influenced by other genetic factors such as synergistic heterozygosity, mosaicism, or epigenetic factors as well as the role of mitochondrial dysfunction with aging.

The high prevalence of NPC heterozygosity (carrier frequency estimated at 1 in 159) (12) implies the existence of unrecognized individuals where early treatment has the potential to reduce or prevent long-term health complications as these heterozygous individuals age. The implications of this are three-fold: first, it warrants screening of NPC heterozygotes for early signs of metabolic or neurodegenerative disorders; second, symptomatic NPC heterozygotes should be identified early, allowing for the possibility of therapeutic intervention with any treatments that prove to be effective for this population; and third, NPC-mechanistic targeted interventions—especially those targeting the final common pathway of neurodegeneration (e.g., lysosomal-mitochondria axis dysfunction) could benefit NPC heterozygotes with subclinical manifestations, particularly those which impact lysosomal or mitochondrial dysfunction (2).

## Conclusion

Recognizing the effects of *NPC1* heterozygosity has profound implications for diagnosis, clinical monitoring, and potential early intervention. More research is needed to understand the impact of pathogenic variants in *NPC1* and *NPC2,* including, but not limited to: first, long-term follow-up over at minimum 1 decade (if not life-long) to systematically assess the dynamics of NPC traits, and second, a genotype–phenotype correlation that to date has been impractical, owing to the size and complexity of the NPC genes. Nevertheless, this might be possible in the future using Artificial Intelligence. Last, but not least, NPC heterozygosity, and heterozygosity for lysosomal storage disorders in general, should be considered in context of epigenetics and genetic modifiers to obtain a comprehensive picture of the potential predisposition to neurodegeneration. This context, together with its potential neuro-modulating, neuroprotective treatments is the basis of the personalized medicine for affected individuals.
